# Directional Phonon Suppression Function as a Tool for the Identification of Ultralow Thermal Conductivity Materials

**DOI:** 10.1038/srep44379

**Published:** 2017-03-24

**Authors:** Giuseppe Romano, Alexie M. Kolpak

**Affiliations:** 1 Department of Mechanical Engineering, Massachusetts Institute of Technology, 77 Massachusetts Avenue, Cambridge, MA, 02139, USA

## Abstract

Boundary-engineering in nanostructures has the potential to dramatically impact the development of materials for high- efficiency conversion of thermal energy directly into electricity. In particular, nanostructuring of semiconductors can lead to strong suppression of heat transport with little degradation of electrical conductivity. Although this combination of material properties is promising for thermoelectric materials, it remains largely unexplored. In this work, we introduce a novel concept, the directional phonon suppression function, to unravel boundary-dominated heat transport in unprecedented detail. Using a combination of density functional theory and the Boltzmann transport equation, we compute this quantity for nanoporous silicon materials. We first compute the thermal conductivity for the case with aligned circular pores, confirming a significant thermal transport degradation with respect to the bulk. Then, by analyzing the information on the directionality of phonon suppression in this system, we identify a new structure of rectangular pores with the same porosity that enables a four-fold decrease in thermal transport with respect to the circular pores. Our results illustrate the utility of the directional phonon suppression function, enabling new avenues for systematic thermal conductivity minimization and potentially accelerating the engineering of next-generation thermoelectric devices.

Understanding heat transport in the presence of nanoscale boundaries is of paramount significance for many applications, including thermoelectrics^1^,^2^, thermal rectifiers^3^ and thermal dissipators^4^. When the dominant phonon mean free path (MFP) Λ approaches the characteristic length scale *L*_*c*_ of a material, classical phonon size effects lead to a decrease in thermal transport^5^. This regime is effectively described by the Knudsen number, defined as the ratio *Kn* = Λ/*L*_*c*_. For example, first-principles calculations of silicon show that half of the heat is carried by phonons with MFPs larger than one micron^6^,^7^, supporting the strong phonon suppression observed in porous materials with microscale pores^8^. In addition, very low thermal conductivities have been measured in many nanostructures, including nanoporous materials^8^,^9^,^10^,^11^,^12^,^13^,^14^, nanowires^15^,^16^ and thin films^17^, corroborating the use of such material systems for thermoelectric applications.

The thermoelectric figure-of-merit in semiconductors is defined as *ZT* = *σS*^2^*T/κ*, where *σ* is the electrical conductivity, *S* is the Seebeck coefficient, *κ* is the lattice thermal conductivity and *T* the temperature. The numerator of ZT (the “power factor”) is generally maximized at relatively high carrier concentrations, so that the average electron MFP is on the order of a few nanometers^18^. Consequently, a properly engineered nanostructure can significantly decreases *κ* with little effect on *σ*, yielding an increase in ZT. Despite many attempts at minimizing thermal transport in nanostructures, however, thermal transport optimization is still largely unexplored, primarily due to practical experimental limitations and a lack of systematic engineering approaches.

In this work, we address the latter by introducing a novel concept, the directional phonon suppression function *S*(Λ, Ω), that describes the suppression of phonons with a given MFP Λ and direction within arbitrary geometries. By taking into account phonons travelling both along straight lines and through multiple phonon-boundary scattering events, *S*(Λ, Ω) turns out to be a powerful tool for tuning thermal transport in complex nanostructures. We employ this approach to optimize thermal transport in Si-based nanoporous materials. We first compute the thermal conductivity, *κ*, of a material system composed of a circular pores in a square lattice, finding significant heat transport degradation with respect to the bulk. Then, we use the information provided by *S*(Λ, Ω) to identify a new structure, based on rectangular pores, that exhibits *κ* as small as 1 Wm^−1^k^−1^, well below the amorphous silicon limit (1.8 Wm^−1^K^−1^)^19^. As our engineering approach can be applied to any combination of material and geometry, it paves the way to high-throughput search of ultra-low thermal conductivity materials.

## Bulk

While the methodology developed in this work is applicable to nanostructures with arbitrary materials and shapes, for the sake of clarity we describe our model specifically in relation to porous Si. The first step for computing heat transport in the presence of nanoscale boundaries is calculation of the thermal conductivity of the corresponding bulk material, which we denote *κ*_*bulk*_. With no loss of generality, throughout the text we consider an isotropic bulk MFP distribution of a thermally isotropic material, *K*_*bulk*_(Λ), which is related to *κ*_*bulk*_ via


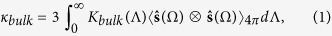


where 〈*f*(Ω)〉_4*π*_ is the angular average (4*π*)^−1^∫_4*π*_*f*(Ω)*d*Ω and 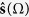 is a versor described in terms of the polar angle *ϕ* and the azimuthal angle *θ*,


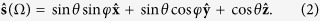


As 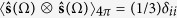, Eq. 1 reduces to the well-known formula 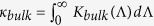. The term *K*_*bulk*_(Λ) is calculated via density functional theory^6^,^7^; for Si at room temperature, we obtain *κ*_*bulk*_ ≈ 155 Wm^−1^k^−1^, in agreement with previous work^20^.

## Macroscopic Limit

**Figure 1:**
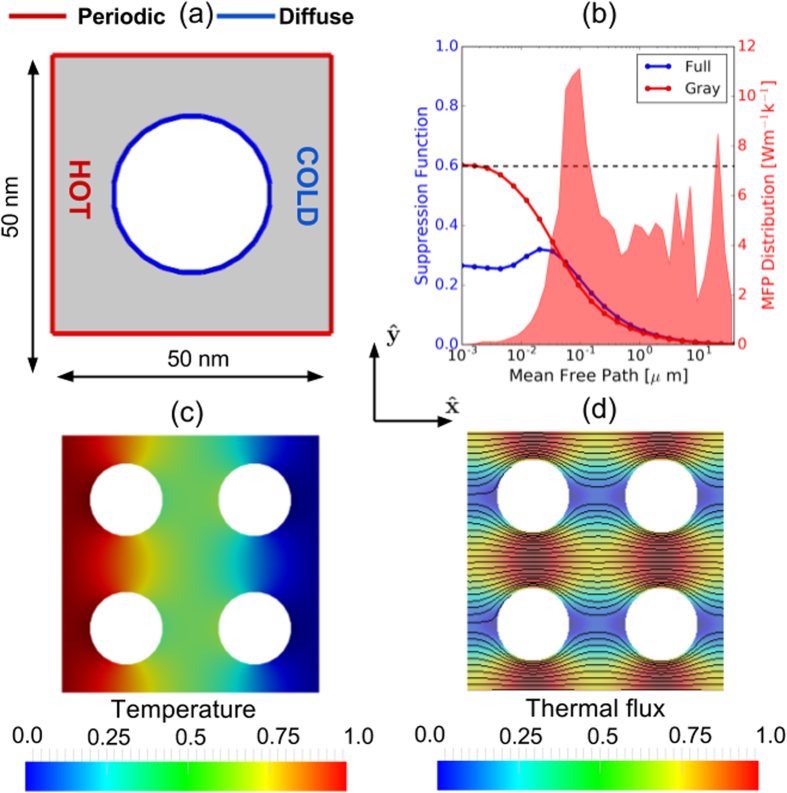
(**a**) Unit-cell comprising a single circular pore. The walls of the pores scatter phonons diffusively. Periodic boundary conditions are applied along both *x*- and *y*- directions. (**b**) Angular average of the directional phonon suppression for different MFPs, for both the gray model and the BTE described by Eq. 5. (**c**) Effective temperature distribution normalized to its maximum value. (**d**) Normalized magnitude of thermal flux superimposed to flux lines.

In porous materials, the volume removal has a degrading effect on heat flow. Furthermore, if the characteristic length *L*_*c*_ of the structure is comparable with the MFPs of heat-carrying phonons, size effects take place and the thermal conductivity is further suppressed. In order to compute the “effective” thermal conductivity *κ* (which is now a scalar) of an array of aligned pores, we identify a square unit-cell with size *L* containing a single pore, which is chosen to be circular, and apply a difference of temperature Δ*T* along 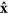, as shown in Fig. 1(a). Periodic boundary conditions are applied at the boundary of the unit cell. We consider the unit cell sizes L = 10 nm and L = 50 nm, keeping the diameter of the pore fixed so that the porosity of the material is *ϕ* = 0.25. Assuming the heat flux, **J**(**r**), is known, we use Fourier’s law, i.e.,


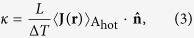


where 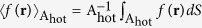 is an average along the surface of the hot contact and 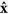 is normal to this surface. We note that the thermal conductivity is now a scalar. For structures in which phonon-size effects are negligible, heat transport can be modelled by the heat diffusion equation, described by the heat flux **J**(**r**) = −*κ*_*bulk*_∇*T*_*L*_(**r**), where *T*_*L*_(**r**) is the spatially dependent lattice temperature, computed by the continuity equation ∇ · **J**(**r**) = 0. Using Eq. 3, we obtain the general expression for the thermal conductivity reduction,


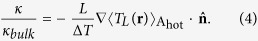


For porous materials with low porosity, macroscopic heat reduction is predicted by the Maxwell-Garnett theory which provides the formula *κ/κ*_*bulk*_ = (1 − *ϕ*)/(1 + *ϕ*) ≈ 93 Wm^−1^k^−1 ^21, in excellent agreement with our finite-volume solver of diffusive heat conduction.

## Directional Phonon Suppression Function

In aligned porous materials, the characteristic length *L*_*c*_ is roughly the pore-pore distance^22^, which in our cases is much shorter than the MFP of most of the dominant phonons in Si^6^, giving rise to significant phonon size effects. In order to take into account these effects, we employ the recently developed MFP-Boltzmann Transport Equation (MFP-BTE)^23^,


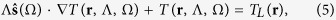


where *T*(**r**, Λ, Ω) is the effective temperature of phonons with MFP Λ and direction 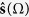 and *T*_*L*_(**r**) is the effective lattice temperature, given by


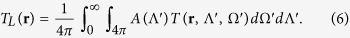


The weights *A*(Λ′) ensure energy conservation and are expressed as


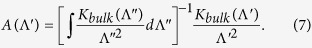


Similarly to ref. 24, the walls of the pores are assumed to scatter phonons diffusively. Further details about the computational approach are reported in the Methods section.

Once Eq. 5 is solved, we include the dependence on Λ and Ω in Eq. 3 to compute the MFP distribution in the nanostructure, i.e.,


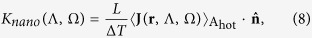


where


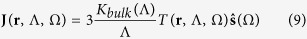


is the thermal flux in the porous material^23^. After substituting Eqs 5, 6, 7, 8, 9 into Eq. 8, we obtain the following relationship


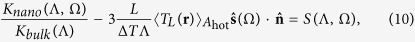


where *S*(Λ, Ω) is the “directional phonon suppression function”, given by


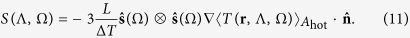


The directional phonon suppression function *S*(Λ, Ω) is central to our work and describes the MFP dependence and directionality of phonon suppression caused by the boundaries. Once *S*(Λ, Ω) is known, the thermal conductivity in the nanostructure is given by


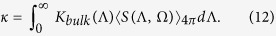


The physical meaning of *S*(Λ, Ω) can be better understood if we apply an angular average to both sides of Eq. 10, which gives


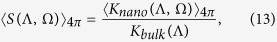


where we used 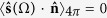. The term 〈*S*(Λ, Ω)〉_4*π*_ is also given by


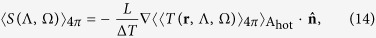


which is the conventional MFP-dependent suppression function. This quantity, also called “boundary scattering”, is proven to be effective in MFP-reconstruction experiments and for understanding heat transport regime^25^,^26^,^27^. We note that the variable Λ is the bulk MFP; any result of this work can be translated in terms of the MFP in the nanostructure, Λ_*nano*_, by means of the transformation Λ_*nano*_ = Λ 〈*S*(Λ, Ω)〉_4*π*_.

## Diffusive Limit

In the following section, we derive the diffusive and ballistic limits of *S*(Λ, Ω) and connect the findings with the heat transport regimes of 〈*S*(Λ, Ω)〉_4*π*_. For short MFPs, *T*(**r**, Λ, Ω) can be expanded up to first-order spherical harmonics as ref. 28


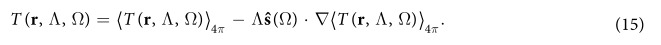


Combining with Eq. 5, multiplying both sides by 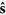 and applying an angular average, this gives^23^,^28^


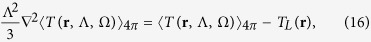


which is a modified heat diffusion equation that takes into account interactions among phonons with different MFPs. After combining Eqs 11, 12, 13, 14, 15, 16, we obtain the diffusive limit of *S*(Λ, Ω),


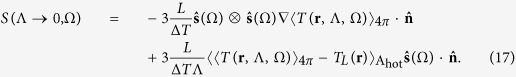


**Figure 2:**
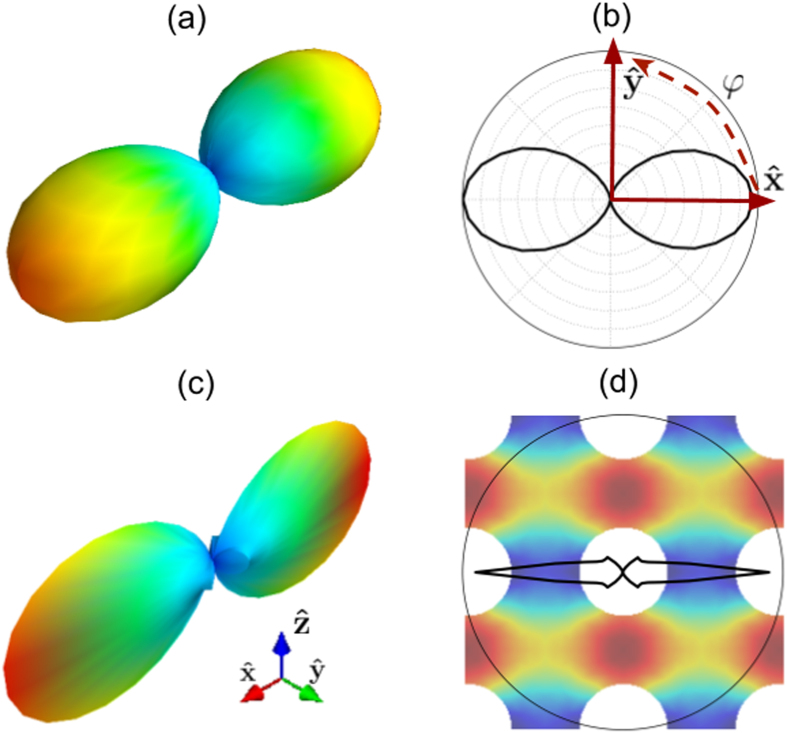
Spherical (**a**) and polar (**b**) representations of *S*(Λ, Ω) for low *Kns*. The two lobes are identical because of the system symmetry. (**c**) Spherical and (**d**) polar representation of *S*(Λ, Ω) for high *Kns*. The two lobes are peaked at *ϕ* = 0 and *ϕ* = *π*. The periodicity is L = 50 nm.

In Eq. 17, the second term represents the fact that the medium is non-gray. In fact, under the gray-medium approximation, where the lattice temperature is *T*_*L*_(**r**, Λ) = 〈*T*(**r**, Λ, Ω)〉_4*π*_, this term vanishes. In this case, phonons are solved independently, leading to a MFP-dependent lattice temperature. For simplicity and with no loss in generality, we now derive the expression of *S*(Λ → 0, Ω) for our nanoporous system within the gray approximation. Under these assumptions, Eq. 16 becomes the Laplacian equation ∇^2^*T*_*L*_(**r**) = 0, *i.e.*, the standard Fourier’s law^29^. By assuming a constant heat flux along the hot contact, the gradient of the lattice temperature is simply 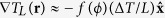 and Eq. 11 then becomes


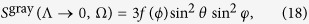


where we used 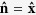. The Cartesian representation of *S*(Λ → 0, Ω), represented by the surface 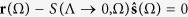, is plotted in Fig. 2(a). We note two lobes, oriented along 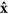 and 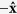, consistently with the fact that both the direction of the applied temperature gradient and the normal of the cold contact are aligned with 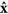. This symmetry can be also seen by the polar representation


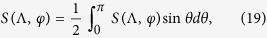


which, when applied to Eq. 17, becomes 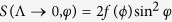, as shown in Fig. 2(b). The diffusive limit of 〈*S*(Λ, Ω)〉_4*π*_ within the gray approximation can be obtained by simply performing an angular average of the polar suppression function, i.e.


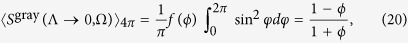


which is the value obtained by Fourier’s law, as shown in Fig. 1(b). However, by using the general expression for non-gray media from Eq. 14, we obtain a lower value for very small MFPs with a peak around 30 nm. This result suggests that the trend obtained by the full MFP-BTE is due to the interaction among phonons with different MFPs. Specifically, the discrepancy in 〈*S*(Λ, Ω)〉_4*π*_ for low-MFP phonons between the gray and the non-gray models arises from the fact that diffusive phonons in Eq. 5 tend to thermalize to an effective temperature (plotted in Fig. 1(c)) that also depends on ballistic phonons.

## Ballistic Limit

We now investigate the ballistic limit of *S*(Λ, Ω). For large MFPs, boundary scattering becomes predominant and *S*(Λ, Ω) starts to depend strongly on the geometry of the material. In this regime, Eq. 5 becomes^5^


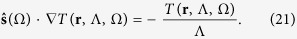


After combining Eqs 11, 12, 13, 14, 15, 16, 17, 18, 19, 20, 21, we have


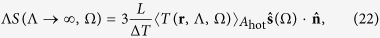


which recovers the well-known behaviour *S*(Λ → ∞, Ω) ∝ Λ^−1^ for the ballistic regime^5^. We note that in this case, the directional suppression function is simply the ratio between the MFP distribution in the nanostructure and that in the bulk, *i.e. K*_*nano*_(Λ → ∞, Ω) = *S*(Λ → ∞, Ω)*K*_*bulk*_(Λ), and so applies to 〈*S*(Λ, Ω)〉_4*π*_. As shown in Fig. 2(c), *S*(Λ, Ω) is pronounced for *ϕ* = 0 and *ϕ* = *π*, whereas it rapidly vanishes for other polar angles. This trend can be explained in terms of the view factor, a geometric parameter that quantifies the possibility of having a direct path between the hot and cold contacts^30^. In porous materials with square pore lattices, most of the heat travels through the spaces between the pores, perpendicular to the applied temperature gradient. The relative contribution of such paths is the view factor. Figure 2(d) superimposes *S*(Λ, Ω) and the material geometry to better elucidate the relationship between the boundary arrangements and phonon suppression. We also note four sub-lobes corresponding to phonons travelling along directions at 45 degrees with respect to the applied temperature gradient, constituting another set of direct paths. For all the other directions, *S*(Λ, Ω) describes Multiple Phonon-Boundary (MPB) scattering. In our case, heat transport arising from MPB is negligible, with most of the heat carried by phonons travelling through direct paths.

## Material Optmization

**Figure 3:**
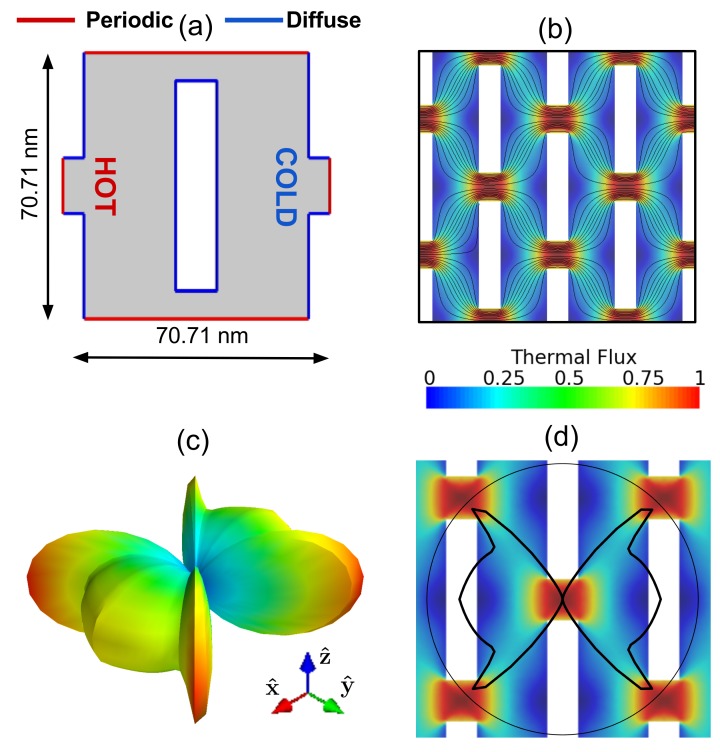
(**a**) Unit-cell of the configuration with staggered rectangular pores. (**b**) Normalized magnitude of thermal flux and flux lines. There are not direct paths from the cold to the hot side. (**c**) Spherical and (**d**) polar representation of *S*(Λ, Ω). All thermal transport arises from multiple phonon boundary scattering.

Using the insights from above, we identify a new nanopore geometry with the same porosity as the circular nanopore case, that has improved properties. This new geometry consists of rectangular pores in a staggered configuration, as shown in Fig. 3(a). The periodicity is larger than the previous case because of the fixed-porosity requirement. From the flux lines plotted in Fig. 3(b), we note that direct paths are absent; thus, phonons scatter multiple times before reaching the cold contact. For low *Kns, S*(Λ, Ω) is similar to that of the case with circular pores, with the size of the lobes determined by the porosity function *f(ϕ*) of the new configuration. Interestingly, for high *Kns, S*(Λ, Ω) has six preferred directions, as shown in Fig. 3(d). The amplitudes of these peaks are significantly smaller than those in Fig. 2(d). Remarkably, the computed *κ* are 4 Wm^−1^k^−1^ and 1 Wm^−1^k^−1^ for L = 50 nm and L = 10 nm, respectively, almost five times smaller than their counterparts with circular pores. We note that, for the case with L = 10 nm, *κ* is smaller than that of amorphous silicon^19^. Finally, as this very low thermal conductivity is obtained with no change in the porosity, the transport of electrons, which travel diffusively, will not be significantly suppressed. As a result, the configuration with staggered rectangular pores has promise as a high-ZT material.

## Conclusions

In summary, we have introduced the directional phonon suppression function, a novel concept that captures the essence of size effects in unprecedented detail. Guided by this new quantity, we have identified a porous structure, based on rectangular pores arranged in a staggered configuration, with ultra-low thermal conductivity and relatively low porosity. Our work furthers the general understanding of boundary-dominated heat transport. In addition, as our approach is suitable for any combinations of material and geometry, this work provides practical guidance for the development of novel, high-efficiency thermoelectric materials.

## Method

The cumulative thermal conductivity requires the calculation of the three-phonon scattering time. These calculations were performed with a 32 × 32 × 32 grid in reciprocal space and a 4 × 4 × 4 supercell. Force constants calculations were performed with a 5 × 5 × 5 supercell. The isotope disorder scattering is included in the calculation. Phonon-related calculations were carried out using ShengBTE^20^. Density functional theory calculations were performed with Quantum Espresso^31^. Using a projected augmented wave (PAW) pseudopotential^32^, with a plane-wave cut-off of 320 meV, and a 11 × 11 × 11 k-point mesh. Phonon size effects were calculated using an in-house code. The spatial domain was discretized using the finite-volume method solved over an unstructured grid with approximately 6000 elements. The solid angle was discretized into 48 polar and 12 azimuthal angles. The material was assumed to be infinite along the *z*-direction. The MFP distribution, assumed to be isotropic, was discretized into 30 MFPs.

## Additional Information

**How to cite this article:** Romano, G. and Kolpak, A. M. Directional Phonon Suppression Function as a Tool for the Identification of Ultra-Low Thermal Conductivity Materials. *Sci. Rep.* **7**, 44379; doi: 10.1038/srep44379 (2017).

**Publisher's note:** Springer Nature remains neutral with regard to jurisdictional claims in published maps and institutional affiliations.
